# Contemporary rates and predictors of fast progression of chronic kidney disease in adults with and without diabetes mellitus

**DOI:** 10.1186/s12882-018-0942-1

**Published:** 2018-06-22

**Authors:** Alan S. Go, Jingrong Yang, Thida C. Tan, Claudia S. Cabrera, Bergur V. Stefansson, Peter J. Greasley, Juan D. Ordonez, for the Kaiser Permanente Northern California CKD Outcomes Study

**Affiliations:** 1 Division of Research, Kaiser Permanente Northern California, 2000 Broadway, Oakland, CA USA; 2 Departments of Epidemiology, Biostatistics and Medicine, University of California, San Francisco, San Francisco, CA USA; 3 Department of Health Research and Policy, Stanford University School of Medicine, Palo Alto, CA USA; 4 Astra-Zeneca, Gothenburg, Sweden; 5 Division of Nephrology, Kaiser Permanente Oakland Medical Center, Oakland, CA USA

**Keywords:** Diabetes, Chronic kidney disease, Progression, Risk factors, Proteinuria, Anemia, Blood pressure

## Abstract

**Background:**

Chronic kidney disease (CKD) is highly prevalent but identification of patients at high risk for fast CKD progression before reaching end-stage renal disease in the short-term has been challenging. Whether factors associated with fast progression vary by diabetes status is also not well understood. We examined a large community-based cohort of adults with CKD to identify predictors of fast progression during the first 2 years of follow-up in the presence or absence of diabetes mellitus.

**Methods:**

Within a large integrated healthcare delivery system in northern California, we identified adults with estimated glomerular filtration rate (eGFR) 30–59 ml/min/1.73 m^2^ by CKD-EPI equation between 2008 and 2010 who had no previous dialysis or renal transplant, who had outpatient serum creatinine values spaced 10–14 months apart and who did not initiate renal replacement therapy, die or disenroll during the first 2 years of follow-up. Through 2012, we calculated the annual rate of change in eGFR and classified patients as fast progressors if they lost > 4 ml/min/1.73 m^2^ per year. We used multivariable logistic regression to identify patient characteristics that were independently associated with fast CKD progression stratified by diabetes status.

**Results:**

We identified 36,195 eligible adults with eGFR 30–59 ml/min/1.73 m^2^ and mean age 73 years, 55% women, 11% black, 12% Asian/Pacific Islander and 36% with diabetes mellitus. During 24-month follow-up, fast progression of CKD occurred in 23.0% of patients with diabetes vs. 15.3% of patients without diabetes. Multivariable predictors of fast CKD progression that were similar by diabetes status included proteinuria, age ≥ 80 years, heart failure, anemia and higher systolic blood pressure. Age 70–79 years, prior ischemic stroke, current or former smoking and lower HDL cholesterol level were also predictive in patients without diabetes, while age 18–49 years was additionally predictive in those with diabetes.

**Conclusions:**

In a large, contemporary population of adults with eGFR 30–59 ml/min/1.73 m^2^, accelerated progression of kidney dysfunction within 2 years affected ~ 1 in 4 patients with diabetes and ~ 1 in 7 without diabetes. Regardless of diabetes status, the strongest independent predictors of fast CKD progression included proteinuria, elevated systolic blood pressure, heart failure and anemia.

## Background

The population burden of chronic kidney disease (CKD) is high, with an estimated prevalence of 15% of U.S. adults in 2007–2012 [1] and 3.3–17.3% in 13 European populations [2]. CKD is independently associated with excess cardiovascular events, death, hospitalization and other adverse outcomes [3]. Stage 3 CKD, defined as an eGFR 30 to 59 ml/min/1.73 m^2^, makes up the largest fraction of CKD patients [1] and carries the highest potential to intervene early and affect the natural history of the disease.

Despite being relatively common, only a small fraction of patients with eGFR 30 to 59 ml/min/1.73 m^2^ progress to end-stage renal disease (ESRD) over their lifetime, with diabetes mellitus being one of the commonly attributed factors [4]. Previous research has focused primarily on the outcome of receipt of renal replacement therapy (chronic dialysis or receipt of renal transplant) that occurs often many years in the future [5–8] and is subject to important biases given that the definition of ESRD is currently based on receiving a procedure [9]. More rapid progression of CKD is associated with worse clinical outcomes (e.g., cardiovascular events and death) independent of current level of eGFR [10–14]. However, predicting which subset of patients are at high risk for progression of their CKD in the short-term is challenging but more clinically and policy relevant when prioritizing resources targeting the highest risk patients to potentially avoid or significantly delay the need for renal replacement therapy and potentially preventing cardiovascular and other adverse outcomes [15]. Prior research on fast progression of CKD has been limited by modest-sized samples, under-representation of racial/ethnic minorities, or being conducted during older eras not reflecting contemporary therapy and management [16–20]. Relatively little is known about whether predictors of the fast CKD progression of CKD varies by diabetes mellitus status, and this could have further clinical and population management implications.

Within a large, diverse cohort of adults with mild-to-moderate CKD, we evaluated contemporary two-year rates and predictors of fast progression of CKD. We hypothesized that there would be higher rates of fast progression in the presence of diabetes and that risk factors may differ based on the presence or absence of diabetes.

## Methods

### Source population

The study was based in KPNC, a large integrated healthcare delivery system currently providing comprehensive care to > 4.4 million members in the San Francisco and greater Bay Area. The KPNC membership is highly representative of the surrounding local and statewide population [21].

### Cohort assembly

We identified all adult (≥ 18 years old) health plan members between January 1, 2008 and December 31, 2010 who had at least one ambulatory, non-emergency department serum creatinine measurement within a regional health plan laboratory and estimated glomerular filtration rate (eGFR) using the CKD-EPI equation [22]. Index date was defined using the first identified serum creatinine measurement during the inception period. We excluded patients whose index eGFR was not between the range of 30 and 59 ml/min/1.72 m^2^. In addition, we excluded patients who had less than 12 months of continuous health plan membership and pharmacy benefit before the index date (defined as the date of the first eGFR test during the inception period) to ensure adequate baseline information. We also excluded those who died, disenrolled or initiated renal replacement therapy (chronic dialysis or receipt of renal transplant) within 24 months after the index date. Deaths were identified from health plan administrative databases (including member proxy reporting), Social Security Administrative vital status files [23], and California state death certificate files [24]. Health plan disenrollment was defined as a continuous membership gap of > 30 days. We also excluded patients without outpatient, non-emergency department eGFR values spaced 10–14 months apart in order to approximate annual measurements during 2 years of follow-up.

### Follow-up and definition of fast progression of CKD

Follow-up occurred through December 31, 2012 for occurrence of CKD progression in eligible patients. We searched only for outpatient, non-emergency department measurements of eGFR for each patient during the first 24 months of follow-up, during which the median number of eGFR measurements was 4 (interquartile range: 3 to 7). We then calculated the annual rate of change in eGFR by fitting a linear regression model where the dependent variable was eGFR consisting of only their baseline and the subsequent two outpatient, non-emergency eGFR values spaced 10–14 months apart during follow-up to ensure consistency across all patients. Fast progression of CKD was defined as experiencing a loss of eGFR > 4 ml/min/1.73 m^2^ per year [25].

### Baseline covariates

Patient demographic characteristics (age, gender and self-reported race/ethnicity) were ascertained from health plan databases. Comorbid conditions were defined based on previously described validated algorithms using relevant information from hospitalization, outpatient and emergency department diagnosis and procedure codes; ambulatory pharmacy dispensing; and laboratory databases [3, 26, 27]. A list of specific definitions, codes and data sources are available on request. Conditions included myocardial infarction, heart failure, valvular heart disease, coronary revascularization (coronary artery bypass surgery, percutaneous coronary intervention), pacemaker placement, atrial fibrillation and/or flutter, ischemic stroke, transient ischemic attack, peripheral artery disease, cardiovascular risk factors (tobacco usage, diabetes mellitus, hypertension, dyslipidemia), other comorbid conditions (cancer, chronic liver disease, chronic lung disease, dementia, depression, extracranial hemorrhage, thyroid disease). We also ascertained data on ambulatory systolic and diastolic blood pressure, heart rate and body mass index, documented proteinuria based on measures of urine dipstick of 1+ or greater [3], as well as the most recent ambulatory level of hemoglobin, LDL cholesterol, HDL cholesterol and serum potassium.

### Statistical approach

All analyses were conducted using SAS statistical software, version 9.3 (Cary, N.C.). Continuous variables were reported as means with standard deviations or medians with interquartile ranges; discrete variables were reported as frequencies and proportions. Given the large sample size, we calculated the difference between the means or proportions of the fast progressors versus non-fast progressors divided by the pooled estimate of the standard deviation, to indicate the standardized difference and considered a value of *d* ≥ 0.10 to represent a meaningful difference [28, 29].

We next calculated the crude risk of fast CKD progression over 24 months by dividing the number of fast progressors by the total number of patients and reported the associated 95% confidence intervals, stratified by baseline status of diabetes mellitus. To initially identify possible independent predictors of fast CKD progression with or without diabetes mellitus at baseline, we performed a two-step model selection process. First, we performed a multivariable logistic regression model stratified by presence or absence of diabetes including all baseline variables in Table 1 as of study entry date, except for specific laboratory measurements in which ≥15% of the patients had no data for that laboratory measurement, as well as any conditions with ≤1% prevalence at baseline. For self-reported race and eligible laboratory measurements, we included a category for unknown. Second, we included in the final multivariable stratified models patient age, gender, race, Hispanic ethnicity, hemoglobin levels and only the subset of other variables that were significantly associated with fast CKD progression using a cutoff of *P* < 0.05 from the initial logistic regression models. Finally, in sensitivity analyses, results were not significantly different if we included the 368 patients who initiated dialysis or received kidney transplantation within the first 2 years of follow-up as fast CKD progressors or if we included or excluded baseline eGFR level, so only results of the main analysis are presented (data not shown).

**Table 1:** Baseline characteristics of adults with index eGFR 30 to 59 ml/min/1.73 m^2^ between January 1, 2008 and December 31, 2012 with annual measurements of kidney function, overall and stratified by those who did or did not experience fast progression of CKD

Characteristic	Not Fast CKD Progression	Fast CKD Progression	*D*-Value^a^
	(*N* = 29,646)	(*N* = 6549)	
Age, yr			
Mean (SD)	73.0 (10.0)	73.3 (10.8)	0.03
Gender, *n* (%)			0.00
Women	16,306 (55.0)	3598 (54.9)	
Men	13,340 (45.0)	2951 (45.1)	
Race/Ethnicity, *n* (%)			
White/European	21,651 (73.0)	4605 (70.3)	0.05
Black/African American	3185 (10.7)	790 (12.1)	0.05
Asian/Pacific Islander	3442 (11.6)	810 (12.4)	0.05
Native American	90 (0.3)	28 (0.4)	0.05
Other	135 (0.5)	45 (0.7)	0.05
Unknown	1143 (3.9)	271 (4.1)	0.05
Known Hispanic ethnicity, *n* (%)	2816 (9.5)	754 (11.5)	0.07
Smoking status, *n* (%)			0.08
Current or former smoker	13,382 (45.1)	3215 (49.1)	
Non-smoker	16,264 (54.9)	3334 (50.9)	
Cardiovascular history, *n* (%)			
Acute myocardial infarction	664 (2.2)	222 (3.4)	0.26
Heart failure	2675 (9.0)	1001 (15.3)	0.36
Hospitalized ischemic stroke	344 (1.2)	105 (1.6)	0.20
Transient ischemic attack	483 (1.6)	128 (2.0)	0.11
Peripheral artery disease	333 (1.1)	103 (1.6)	0.21
Mitral and/or aortic valvular disease	1973 (6.7)	479 (7.3)	0.06
Atrial fibrillation and/or flutter	3086 (10.4)	830 (12.7)	0.13
Cardiac procedure history, *n* (%)			
Coronary artery bypass graft surgery	422 (1.4)	112 (1.7)	0.11
Percutaneous coronary intervention	820 (2.8)	233 (3.6)	0.16
Pacemaker	310 (1.0)	106 (1.6)	0.27
Medical history, *n* (%)			
Diabetes mellitus	10,139 (34.2)	3035 (46.3)	0.31
Proteinuria	3032 (10.2)	1458 (22.3)	0.56
Hypertension	24,838 (83.8)	5772 (88.1)	0.22
Diagnosed dementia	685 (2.3)	187 (2.9)	0.13
Diagnosed depression	3899 (13.2)	903 (13.8)	0.03
Dyslipidemia	22,157 (74.7)	5111 (78.0)	0.11
Chronic liver disease	580 (2.0)	156 (2.4)	0.12
Chronic lung disease	6299 (21.2)	1546 (23.6)	0.08
Hyperthyroidism	865 (2.9)	185 (2.8)	0.02
Hypothyroidism	5555 (18.7)	1216 (18.6)	0.01
Systemic cancer	2042 (6.9)	451 (6.9)	0.00
Extracranial hemorrhage	553 (1.9)	157 (2.4)	0.16
Body mass index, kg/m^2^, *n* (%)			0.03
< 18.5	247 (0.8)	66 (1.0)	
18.5–24.9	6664 (22.5)	1418 (21.7)	
25.0–29.9	10,290 (34.7)	2180 (33.3)	
30.0–39.9	9038 (30.5)	2067 (31.6)	
≥ 40.0	1497 (5.0)	448 (6.8)	
Unknown	1910 (6.4)	370 (5.6)	
Systolic blood pressure, mmHg			
Mean (SD)	129.0 (16.5)	132.9 (18.7)	0.23
Diastolic blood pressure, mmHg			
Mean (SD)	70.9 (10.4)	71.1 (11.1)	0.02
Baseline medication use, *n* (%)			
Angiotensin-converting enzyme inhibitor	13,634 (46.0)	3252 (49.7)	0.09
Angiotensin II receptor blocker	4335 (14.6)	1216 (18.6)	0.17
Diuretic	17,136 (57.8)	3997 (61.0)	0.08
Loop	4470 (15.1)	1502 (22.9)	0.31
Thiazide	13,377 (45.1)	2739 (41.8)	0.08
β-blocker	15,256 (51.5)	3759 (57.4)	0.15
Calcium channel blocker	7607 (25.7)	2110 (32.2)	0.19
Alpha-blocker	3927 (13.2)	1023 (15.6)	0.12
Aldosterone receptor antagonist	691 (2.3)	191 (2.9)	0.14
Isosorbide dinitrate + hydralazine	66 (0.2)	48 (0.7)	0.73
Hydralazine	903 (3.0)	374 (5.7)	0.40
Antiarrhythmic	704 (2.4)	189 (2.9)	0.12
Nitrate	1478 (5.0)	479 (7.3)	0.25
Digoxin	1072 (3.6)	303 (4.6)	0.16
Statin	18,442 (62.2)	4342 (66.3)	0.11
Other lipid-lowering agent	1880 (6.3)	505 (7.7)	0.13
Non-steroidal anti-inflammatory drug	4028 (13.6)	803 (12.3)	0.07
Antiplatelet agent	1535 (5.2)	422 (6.4)	0.14
Diabetic therapy	7945 (26.8)	2541 (38.8)	0.33
Erythropoietin	160 (0.5)	81 (1.2)	0.51
Baseline laboratory values			
CKD-EPI eGFR, ml/min/1.73m^2^			0.07
45–59	20,263 (68.3)	4675 (71.4)	
30–44	9383 (31.7)	1874 (28.6)	
Hemoglobin, g/dL, *n* (%)			0.08
≥ 13.0	15,328 (51.7)	2706 (41.3)	
12.0–12.9	5160 (17.4)	1326 (20.2)	
11.0–11.9	2656 (9.0)	937 (14.3)	
10.0–10.9	994 (3.4)	383 (5.8)	
9.0–9.9	315 (1.1)	117 (1.8)	
< 9.0	125 (0.4)	42 (0.6)	
Unknown	5068 (17.1)	1038 (15.8)	
Low density lipoprotein cholesterol, mg/dL			
Mean (SD)	97.4 (33.1)	94.0 (33.6)	0.10
Median (IQR)	92.0 (75.0–116.0)	88.0 (71.0–111.0)	
Range	12.0–531.0	19.0–339.0	
Missing, *n* (%)	2997 (10.1)	659 (10.1)	
High density lipoprotein cholesterol, mg/dL			
Mean (SD)	49.7 (14.2)	48.3 (13.8)	0.10
Median (IQR)	47.0 (40.0–57.0)	46.0 (39.0–55.0)	
Range	10.0–153.0	4.0–178.0	
Missing, *n* (%)	3560 (12.0)	807 (12.3)	
Serum potassium, mmol/L			
Mean (SD)	4.5 (0.5)	4.5 (0.5)	0.03
Median (IQR)	4.5 (4.2–4.8)	4.5 (4.2–4.8)	
Range	2.1–6.7	2.6–7.3	
Missing, *n* (%)	2761 (9.3)	376.7)	

^a^ Represents the Cohen’s D-value, calculated by dividing the difference of the group means by the standard deviation, with a *D*-value ≥ 0.10 considered a meaningful difference

Using the final models, we also examined model accuracy by evaluating calibration (i.e., how correctly the model predicts absolute risk) as well as discrimination (i.e., the ability of the model to identify higher vs. lower risk patients) to provide insights into the clinical utility of our findings [30].

## Results

### Cohort assembly and baseline characteristics

We identified 36,195 eligible adults with index eGFR 30 to 59 ml/min/1.73 m^2^ between January 1, 2008 and December 31, 2010, with 36% of patients having diabetes mellitus. In the overall cohort, mean age was 73.0 years with 55% being women, 11% black/African American, 12% Asian/Pacific Islander and 10% Hispanic (Table 1). Overall, 85% had hypertension and three quarters of patients had known dyslipidemia (Table 1).

**Fig. 1:**
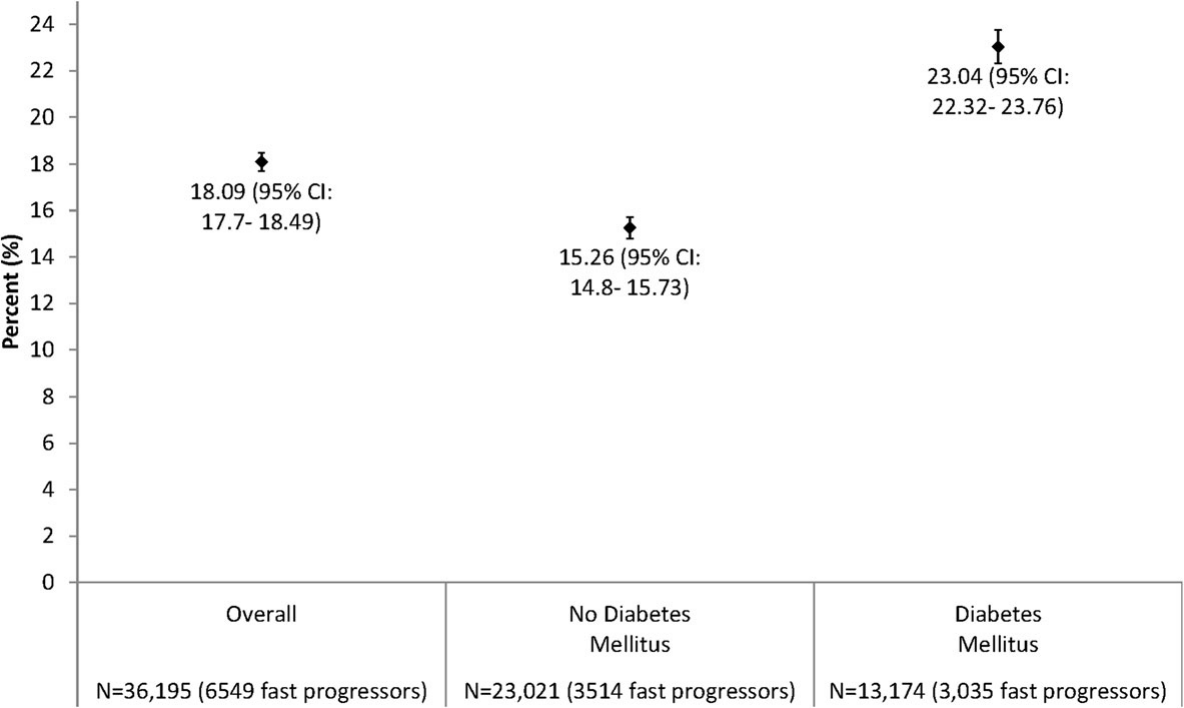
Risk of fast progression of CKD over 24 months among adults with eGFR 30 to 59 ml/min/1.73 m^2^, overall and stratified by baseline status of diabetes mellitus

During 2 years of follow-up, the median annual change in renal function in the overall cohort was 0.04 (interquartile range − 2.89 to 3.08) ml/min/1.73 m^2^. A total of 6549 patients (18.1%) experienced fast decline in kidney function over 24 months, with a higher crude risk in those with vs. without diabetes mellitus (Fig. 1). Fast progressors were more likely to have prior cardiovascular diseases (acute myocardial infarction, coronary artery bypass graft surgery, percutaneous coronary intervention, heart failure, atrial fibrillation/atrial flutter, pacemaker) as well as prior ischemic stroke, transient ischemic attack, peripheral artery disease, proteinuria, diabetes mellitus, hypertension, dementia, dyslipidemia, chronic liver disease, and thyroid disease (Table 1). Patients who experienced fast progression were also more likely to have higher mean systolic blood pressure than those who were not fast progressors. In addition, fast progressors were more likely to be receiving angiotensin II receptor blockers, loop diuretics, β-blockers, calcium channel blockers, alpha blockers, aldosterone receptor antagonists, isosorbide dinitrate/hydralazine, hydralazine, antiarrhythmic therapy, nitrates, digoxin, statin, other lipid lowering therapies, antiplatelet agents, diabetic therapy and erythropoietin (Table 1).

### Multivariable predictors of fast CKD progression by diabetes status

Given our hypothesis that predictors of being a fast progressor may vary in the presence or absence of diabetes, we performed models stratified by baseline diabetes status.

**Fig. 2:**
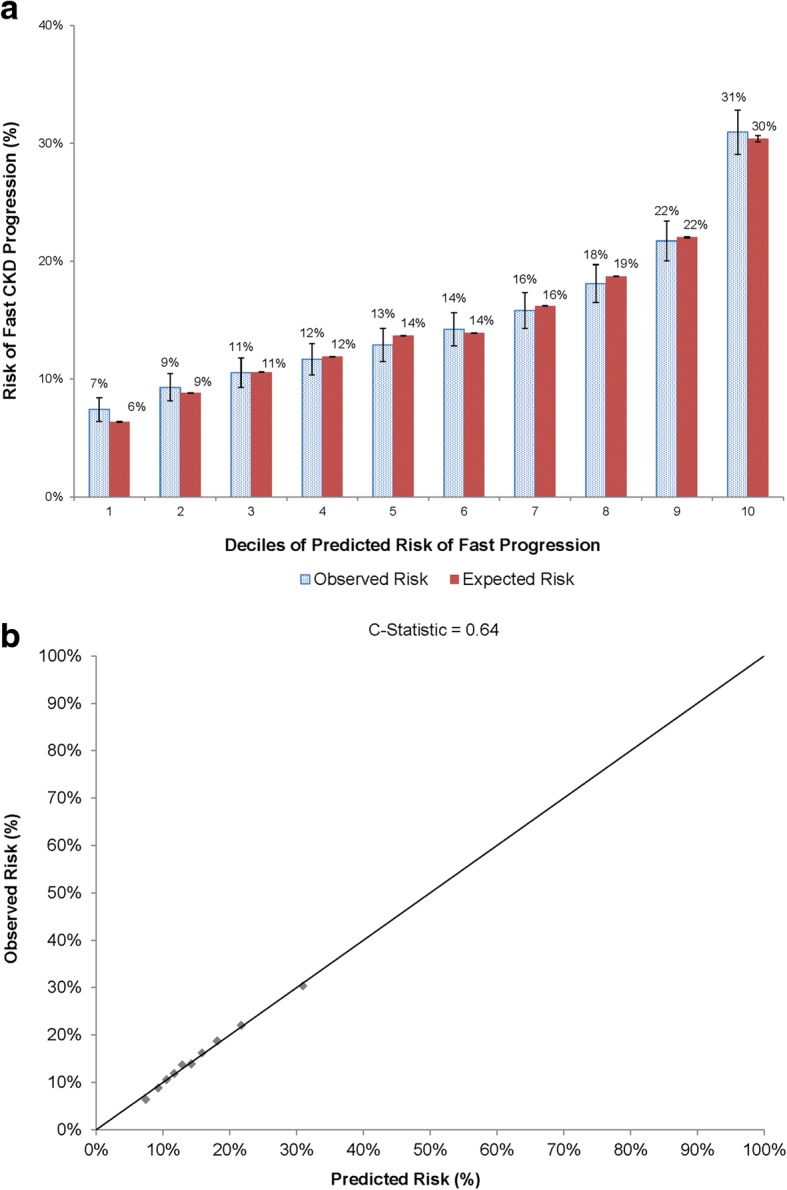
Accuracy of predictive model for fast CKD progression over 2 years in patients with eGFR 30 to 59 ml/min/1.73 m^2^ and no diabetes mellitus at entry, based on (**a**) comparison of observed vs. predicted risks within deciles of predicted risk and (**b**) model calibration across the spectrum of observed risk

In patients without diabetes mellitus at entry, multivariable predictors of fast CKD progression included age ≥ 70 years, heart failure, prior ischemic stroke, prior pacemaker implantation, proteinuria, higher entry level of eGFR, lower hemoglobin levels and HDL cholesterol < 50 mg/dL (Table 2). There was a graded increased independent risk of fast progression with systolic blood pressure above 120 mmHg. Model calibration was outstanding across a range of absolute risk of fast CKD progression over 2 years (Fig. 2a and b), while model discrimination was moderate (c statistic 0.64).

**Table 2:** Selected multivariable predictors of fast CKD progression over 24 months among adults with eGFR 30 to 59 ml/min/1.73 m^2^ stratified by baseline diabetes status

Characteristic	Adjusted^a^ Odds Ratio (95% Confidence Interval) for Fast Progression in Adults with Stage 3 CKD and no Diabetes *N* = 23,021	Adjusted^b^ Odds Ratio (95% Confidence Interval) for Fast Progression in Adults with Stage 3 CKD and Diabetes *N* = 13,174
Age group at study entry, yr		
18–49	1.16 (0.91–1.48)	1.60 (1.22–2.10)
50–59	0.72 (0.61–0.86)	1.16 (1.00–1.34)
60–69	0.81 (0.73–0.90)	1.03 (0.92–1.14)
70–79	Reference	Reference
≥ 80	1.25 (1.14–1.36)	1.16 (1.03–1.30)
Male vs. female gender	1.00 (0.92–1.08)	0.99 (0.91–1.08)
Race		
White/European	Reference	Reference
Black/African American	1.02 (0.90–1.16)	1.00 (0.88–1.14)
Asian/Pacific Islander	1.00 (0.88–1.15)	1.02 (0.91–1.15)
Native American	1.57 (0.81–3.04)	1.22 (0.67–2.20)
Other	1.26 (0.68–2.34)	1.24 (0.81–1.92)
Unknown	0.88 (0.69–1.11)	1.15 (0.94–1.42)
Hispanic ethnicity	1.05 (0.90–1.21)	1.03 (0.90–1.18)
Current/former smoking	1.21 (1.12–1.30)	–
Medical history		
Heart failure	1.77 (1.57–1.99)	1.58 (1.40–1.79)
Ischemic stroke	1.50 (1.11–2.04)	–
Pacemaker	1.43 (1.06–1.92)	–
Proteinuria	1.86 (1.65–2.10)	2.31 (2.10–2.55)
Valvular heart disease	–	0.86 (0.71–1.03)
Systolic blood pressure, mmHg		
≤ 120	Reference	Reference
121–129	1.17 (1.04–1.30)	1.14 (1.01–1.29)
130–139	1.28 (1.15–1.41)	1.23 (1.08–1.39)
140–159	1.44 (1.28–1.62)	1.47 (1.29–1.68)
160–179	2.07 (1.75–2.46)	1.99 (1.64–2.42)
≥ 180	2.42 (1.80–3.24)	2.21 (1.59–3.08)
Unknown	1.12 (0.88–1.43)	1.20 (0.94–1.53)
Laboratory values		
Hemoglobin, g/dL		
≥ 13.0	Reference	Reference
12.0–12.9	1.38 (1.25–1.53)	1.39 (1.23–1.56)
11.0–11.9	2.02 (1.78–2.29)	1.63 (1.43–1.87)
10.0–10.9	2.09 (1.73–2.54)	1.76 (1.47–2.11)
9.0–9.9	1.46 (1.02–2.10)	2.09 (1.55–2.81)
< 9.0	1.44 (0.85–2.43)	2.15 (1.26–3.68)
Unknown	1.13 (1.01–1.26)	1.15 (1.02–1.30)
HDL cholesterol, mg/dL		
≥ 60	Reference	–
50–59	1.09 (0.97–1.23)	–
40–49	1.16 (1.04–1.30)	–
35–39	1.25 (1.08–1.45)	–
< 35	1.27 (1.07–1.50)	–
Unknown	1.20 (1.07–1.36)	–
LDL cholesterol, mg/dL		
≥ 200	–	Reference
160–199	–	1.73 (1.06–2.84)
130–159	–	1.07 (0.80–1.44)
100–129	–	1.10 (0.89–1.35)
70–99	–	1.03 (0.90–1.17)
< 70	–	0.93 (0.84–1.02)
Unknown	–	1.07 (0.82–1.39)

^a^Adjusted also for baseline level of eGFR and receipt of angiotensin II receptor blockers and calcium channel blockers at entry

^b^Adjusted also for baseline level of eGFR and receipt of diuretics, calcium channel blockers and hydralazine at entry

**Fig. 3:**
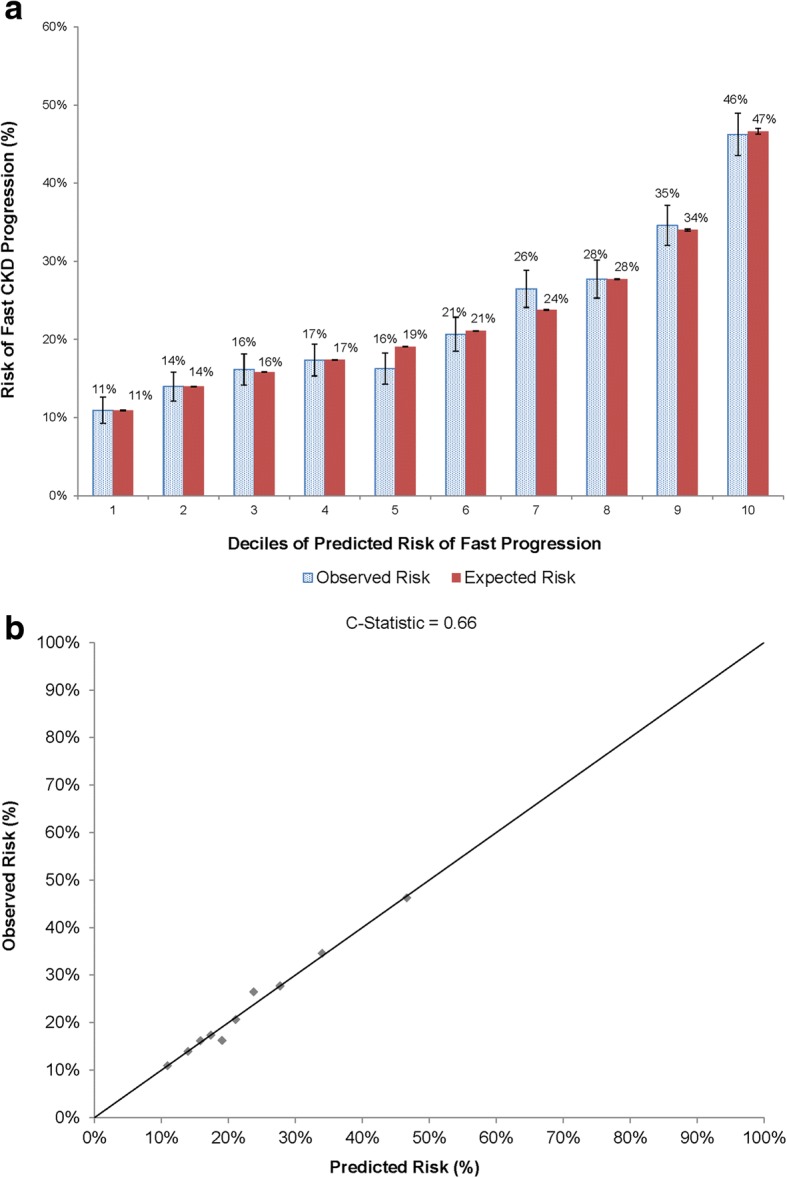
Accuracy of predictive model for fast CKD progression over 2 years in patients with eGFR 30 to 59 ml/min/1.73 m^2^ and diabetes mellitus at entry, based on (**a**) comparison of observed vs. predicted risks within deciles of predicted risk and (**b**) model calibration across the spectrum of observed risk

In patients with diabetes mellitus at entry, results were largely similar to that found in non-diabetic patients with regards to heart failure, known proteinuria, higher entry level of eGFR, and the increased risk of fast progression with lower hemoglobin levels and higher systolic blood pressure, while there was a different association for age in which age 18–49 and ≥ 80 years were associated with higher adjusted odds of fast progression (Table 2). However, there was no significant multivariable association noted for current or former cigarette smoking, prior ischemic stroke, prior pacemaker implantation or lower HDL cholesterol level (Table 2). Similar to results in patients without diabetes, model calibration was outstanding across an even broader range of absolute risk of fast CKD progression over 2 years (Fig. 3a and b), while model discrimination was moderate (c statistic 0.66).

## Discussion

There has been a growing appreciation that a significant proportion of patients with mild-to-moderate CKD do not progress in a predictable, slow linear pattern, with some patients either not progressing and others having a much faster decline in kidney function [31]. Identifying the subset of mild-to-moderate CKD patients who will subsequently experience fast CKD progression in the short-term has important clinical implications as they may benefit from more intensive monitoring and intervention. To address this, we examined a large, diverse community-based cohort with eGFR 30 to 59 ml/min/1.73 m^2^ and found that 23% of these patients with diabetes and 15% of those without experienced fast CKD progression, and that the significant multivariable factors regardless of diabetes status included age ≥ 80 years, proteinuria, higher systolic blood pressure, lower hemoglobin level, and known heart failure, even after accounting for initial level of eGFR and receipt of cardiovascular and renal-related medications.

While CKD “progression” has been investigated in various ways, the large majority of studies focused on the end point of ESRD defined as the receipt of chronic dialysis or renal transplantation [5–8]. Other studies have used varying definitions in the absolute or relative amount of drop in eGFR or reaching a particular level of eGFR [32]. While these are all potentially relevant outcomes, existing studies frequently use long time horizons and do not necessarily address the rate of change, which may be more important clinically to identify the subset of patients who are losing kidney function quickly in the short-term and may benefit from earlier, more frequent surveillance and intervention. Toward that end, our cohort of patients with Stage 3 CKD who had annual outpatient measurements of eGFR during 2 years of follow-up provides important insights into the rate of fast CKD progression and associated patient characteristics.

Our finding that documented proteinuria, a reflection of underlying structural kidney damage, is a strong predictor of short-term, fast CKD progression is consistent with other studies that have examined the development of ESRD or significant reductions in the absolute level of eGFR over a longer period of follow-up [5–8, 32].

In patients with diabetes, the youngest patients with CKD were also more likely to experience fast progression after accounting for other potential risk factors and use of cardioprotective and renoprotective therapies, which is consistent with other studies [32] and may reflect a difference in underlying cause(s) of CKD. In contrast, among patients without diabetes, those aged 50–69 years old were less likely than those aged 70–79 years old to have fast CKD progression. Patients ≥80 years were more likely to experience fast progression regardless of diabetes status. We found that current or former cigarette smoking was associated with fast CKD progression in the short-term among patients without diabetes, which is consistent with results from the Cardiovascular Health Study cohort that showed a 31% relative increase in the likelihood of a serum creatinine increase ≥0.3 mg/dL over 3 years [33]. Furthermore, among 1906 participants in the Italian Longitudinal Study on Aging (ISA) cohort, current smoking > 20 cigarettes/day was associated with a more than twofold higher odds (adjusted odds ratio 2.29, 95% CI:1.00–5.27) of an increase in serum creatinine > 0.3 mg/dL during mean 3.6-year follow-up [34]. However, conflicting results exist about the relationship between smoking history and longer-term endpoints such as the need for renal replacement therapy [35, 36].

Higher systolic blood pressure is a well-documented risk factor of development of ESRD [35, 37] or a 50% reduction in baseline eGFR in patients with CKD [38], but fewer studies have examined the influence of blood pressure on rate of change of eGFR. Among 220 participants with eGFR 30–59 ml/min/1.73 m^2^ enrolled in the Multi-Ethnic Study of Atherosclerosis (MESA), having a systolic blood pressure ≥ 130 mmHg was associated with a more than twofold higher adjusted odds (odds ratio 2.29, 95% CI:1.20–4.37) of CKD progression, defined as > 2.5 ml/min/1.73 m^2^ decline in eGFR over 5 years of follow-up when estimating GFR using combined serum creatinine and cystatin C data [39]. Our study supports and extends these findings in a much larger cohort showing a graded increased risk of fast CKD progression with systolic blood pressure > 120 mmHg even after additional adjustment for receipt of different antihypertensive agents.

Lower hemoglobin levels below 13.0 g/dL were also associated with a higher risk of fast progression. These results are consistent with an analysis of 6100 adults with systolic heart failure showing that a hematocrit < 36% (hemoglobin of approximately < 12.0 g/dL) was associated with a higher adjusted odds (odds ratio 1.30, 95% CI:1.18–1.45) of rapid decline in kidney function (defined as ≥6 ml/min/1.73 m^2^ decrease in eGFR per year) and was more prominent in those with CKD (adjusted odds ratio 1.71, 95% CI:1.43–2.05) [20]. In addition, hemoglobin levels were lower among 117 Stage 3 CKD patients who progressed (eGFR decline > 3 ml/min/1.73 m^2^ per year over 4 years) compared with 364 Stage 3 CKD patients considered non-progressors (< 1 ml/min/1.73 m^2^ per year over 4 years) [40].

There have been conflicting data about the association between different lipoproteins and the risks of ESRD or significant loss of kidney function in adults with CKD. For example, in the Chronic Renal Insufficiency Cohort (CRIC) Study, none of the lipoproteins studied (total, LDL, HDL, VLDL, Lp(a), apoA-I, apoB) were independently associated with development of ESRD or > 50% reduction in baseline eGFR [41]. In contrast, among a Japanese population with CKD [42], the highest two quartiles of triglyceride-to-HDL cholesterol ratio were associated with rapid decline (defined as > 5 ml/min/1.73 m^2^ reduction per year) compared with the lowest quartile, which supports our observation that a lower HDL level was associated with a graded, increased risk of fast CKD progression in the subset of patients without diabetes.

Despite evaluating a wide range of candidate predictors of fast progression of CKD, our model discrimination was moderate for models in patients with (c statistic 0.66) or without (c statistic 0.64) diabetes which is in contrast to models of initiation of renal replacement therapy which achieve much higher c statistics (0.84–0.90) [5, 8], depending on the severity of the CKD population studied and time horizon used. This highlights the challenge of accurately discriminating which subset of patients starting at an eGFR 30 to 59 ml/min/1.73 m^2^ will experience fast progression of CKD and the need for investigation of additional factors influencing this risk to potentially intervene earlier in the process to delay the progression to ESRD. Importantly, however, model calibration (i.e., the ability to correctly estimate absolute risk of fast CKD progression over 2 years) was outstanding in patients with and without diabetes at entry, which highlights the potential clinical value in providing prognostic information to patients with stage 3 CKD (Figs. 2 and 3).

The study was strengthened by inclusion of a large, racially/ethnically diverse sample of adults with mild-to-moderate CKD who had approximately annual outpatient, non-emergency department measurements of serum creatinine from baseline through the first 2 years of follow-up. This allowed us to examine annual rate of eGFR decline and to identify the subgroup of patients who experienced fast CKD progression. All relevant diagnoses, procedures, test results and treatments were comprehensively captured through a state-of-the-art electronic medical record system that facilitated assessment of relevant patient characteristics which may predict fast CKD progression. Our study also had some limitations. For each patient, we calculated an average annual rate of eGFR decline estimated using linear regression, but patients may experience non-linear patterns of change in kidney function not reflected using this approach. Since this was a retrospective study of patients receiving clinically obtained measures of eGFR (rather than a structured research protocol with annual assessment of kidney function), the findings may not apply to all patients with eGFR 30–59 ml/min/1.73 m^2^. We also used estimated GFR rather than measured GFR which may lead to some misclassification [43]. Similarly, results may not be completely generalizable to other clinical practice settings or to uninsured patients who may experience a different intensity of treatment or follow-up. Even though we examined a wide range of patient characteristics, we cannot rule out residual confounding affecting our model results. We were unable to comprehensively identify exposure to nephrotoxic insults or episodes of acute kidney injury from available data. Systematic information on other selected biomarkers that have been associated with different measures of CKD progression were also not available (e.g., urinary phosphorus excretion [44], serum calcium and phosphorus [40], serum bicarbonate [40, 45], NT-proBNP [19], or troponin T [19]).

## Conclusions

In sum, within a large, contemporary population of adults with mild-to-moderate CKD, accelerated progression of kidney dysfunction within 2 years affected ~ 1 in 4 patients with diabetes and ~ 1 in 7 without diabetes. Regardless of diabetes status, the independent predictors of fast CKD progression included proteinuria, elevated systolic blood pressure, heart failure, anemia, and age ≥ 80 years. Age 18–49 years was also a predictor in those with diabetes, while additional predictors in patients without diabetes included smoking history, prior ischemic stroke, prior pacemaker implantation, and HDL cholesterol < 60 mg/dL. These findings may help in identifying the subset of patients who may benefit from more frequent monitoring of eGFR to identify fast CKD progression or proteinuria and subsequent intervention to potentially retard further loss of kidney function and other potentially associated adverse clinical outcomes such as cardiovascular events.

## Abbreviations

CKD: Chronic kidney disease
eGFR: Estimated glomerular filtration rate
ESRD: End-stage renal disease
KPNC: Kaiser Permanente Northern California
